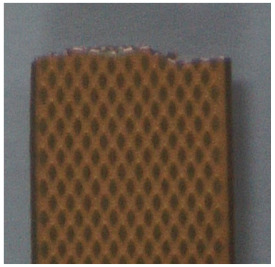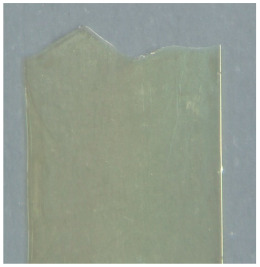# Mechanical Characterization and Analysis of Different-Type Polyimide Feedthroughs Based on Tensile Test and FEM Simulation for an Implantable Package

**DOI:** 10.3390/mi13081295

**Published:** 2022-08-11

**Authors:** Seonho Seok, HyungDal Park, Yong-Jun Kim, Jinseok Kim

**Affiliations:** 1 Center for Nanoscience and Nanotechnology (C2N), University-Paris-Saclay, 91400 Orsay, France; 2 Center for Bionics, Korea Institute of Science and Technology (KIST), Seongbuk-gu, Seoul 02792, Korea; 3 School of Mechanical Engineering, Yonsei University, Seodaemun-gu, Seoul 03722, Korea

**Keywords:** mechanical characterization, polymer feedthroughs, FEM (finite element method), implantable package, tensile test

## Abstract

This paper presents the mechanical behaviors of different types of polyimide feedthroughs that are frequently used for implantable polymer encapsulation. Implantable packages of electronic devices often comprise circuits mounted on printed circuit boards (PCBs) encapsulated in a biocompatible polymer material, with input/output feedthroughs for electrical interconnections. The feedthroughs are regarded as essential elements of the reliability of the package since they create inevitable interfaces with the encapsulation materials. Flexible materials are frequently used for feedthroughs owing to their ease of manufacturing; thus, their mechanical properties are crucial as they directly interact with parts of the human body, such as the brain and neurons. For this purpose, tensile tests were performed to characterize the mechanical properties of flexible PCBs (FPCBs) and photosensitive polyimides (PSPIs). Commercial FPCBs and homemade PSPIs of two different thicknesses were subjected to tensile tests for mechanical characterization. The FPCBs showed typical stress–strain curves, while the PSPIs showed brittleness or strain hardening depending on the thickness. The material properties extracted from the tensile tests were used for explicit modeling using the finite element method (FEM) and simulations to assess mechanical behaviors, such as necking and strain hardening.

## 1. Introduction

Biomedical implants and devices are expected to be in high demand in the future as they enhance the quality of human life [1,2]. Such implants or devices must be suited for long-term use in the human body to avoid side effects. Therefore, these devices should be appropriately packaged before installation in the human body. Biocompatible packaging aims to provide protection for the implanted electronic device so that it can tolerate the harsh biological environment and extend the lifetime of the implanted device [3,4,5]. In existing commercial medical devices such as pacemakers, titanium (Ti) boxes are often used to ensure hermetic and biocompatible packaging of the microelectronic device [6]. While Ti boxes are well-known hermetic implant packaging materials, they evoke pronounced foreign body reactions (FBRs) upon implantation, resulting in encapsulation by a thick layer of fibrous tissue that might decrease the sensitivity of the implanted sensor [7]. Furthermore, mechanical mismatches between the Ti box and local tissues may cause chronic discomfort to the patient.

To address these drawbacks of existing Ti boxes, advanced biocompatible packaging has been proposed by mainly two approaches, namely polymer encapsulation of the conventional circuit boards and chip-scale packaging [3]. Polymer materials have been studied extensively, with particular attention on polyimides, parylene, and silicones [8,9,10]. Such polymer materials, preferably biocompatible ones, are used to seal the printed circuit boards (PCBs) or silicon chips to ensure the biocompatibility of the electronic systems [11,12,13]. Consequently, different feedthroughs based on electrical, chemical, mechanical, or optical methods are inevitably introduced for the implanted devices to interact with their surrounding biological media.

The reliability of a biocompatible package highly depends on the material properties of the sealing polymer, such as its mechanical elasticity, chemical resistance, and adhesion. The permeability of the polymer itself can be controlled or improved by adding additional passivation dielectric layers or multiple polymer/dielectric layers [14,15]. Moreover, it has been reported that the main cause of the failure of polymer biocompatible packages is the lack of perfect adhesion between the polymer and device surface [16,17,18,19]. Feedthroughs are typically composed of metal electrodes attached to polymer materials and are frequently used for advanced neural recording. Adhesion of the electrode to the supporting polymer substrate is a critical factor for the long-term reliability of a neural recording system. To date, many reports have focused on the quality of the encapsulation polymer materials, their adhesion to the packaged device, and the mechanical integrity of the electrode with the polymer substrate [20,21,22,23]. The stress–strain behaviors of polymeric materials depend on various parameters, such as molecular characteristics, microstructures, strain rates, and temperatures. Further, high-strain behaviors and failures of the different kinds of polymers can be estimated by tensile tests [24].

In this study, the mechanical behaviors of polyimide feedthroughs were characterized on the basis of tensile tests of implantable packages. The objective of the tensile test was to determine the mechanical behaviors of the polyimide feedthroughs when large amounts of strain were applied. Concepts related to the implantable package under study as well as the test sample preparation procedure are described in Section 2. The tensile test methods and results are explained in Section 3. Section 4 presents the FEM modeling and simulation of the tensile tests of thin polymer films using the material properties extracted from characterization, and Section 5 presents the conclusions of the study.

## 2. Concept of Implantable Package

**Figure 1:**
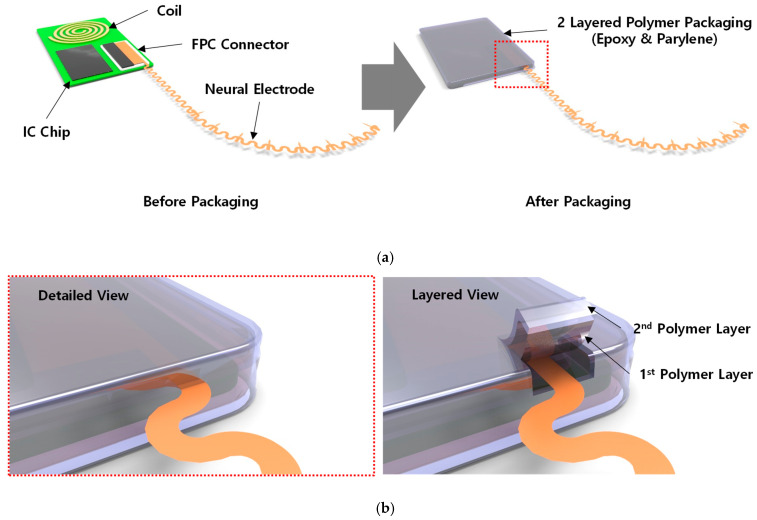
Concept of implantable biocompatible packaging. (**a**) Overall view. (**b**) Enlarged view of the feedthrough of the layered polymer package (dotted rectangle in red color in (**a**)).

The concept of an implantable biocompatible package is shown in Figure 1; it consists of a photosensitive polyimide (PSPI)-based neural probe, Si chips on a PCB, and the flexible PCB (FPCB) cable. The Si chips are affixed and wire-bonded to the PCBs, followed by encapsulation in an epoxy polymer with a parylene overcoat to achieve biocompatibility. The neural probe is fabricated from a biocompatible PSPI material that has a thickness of a few micrometers and a length of a few millimeters. The FPCB cable is used to deliver amplified neural signals to subsequent electronic modules, such as a wireless communication device. The reliability of the biocompatible package depends on the quality of the epoxy encapsulation with the parylene overcoat, adhesion between the epoxy polymer and PSPI or FPCB, and mechanical stabilities of the PSPI and FPCB cables. The quality of the encapsulation polymer is evaluated by the soaking test, and the adhesion between the encapsulation polymer and feedthrough can be measured through the tensile and shear tests [25]. However, the mechanical characteristics of the flexible cables have not been studied previously, which may be important during their operation in the human body.

**Figure 2:**
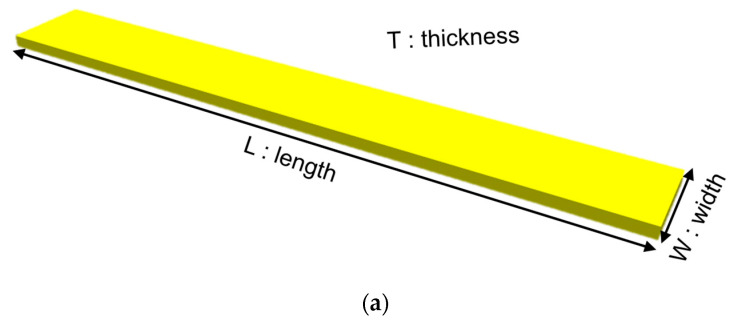

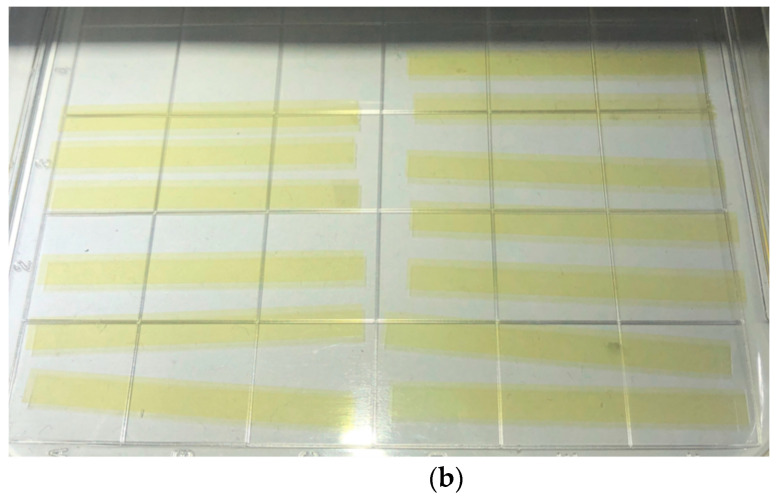
Test samples. (**a**) Dimensions of the rectangular test sample. (**b**) Released PSPI samples placed on PDMS.

The rectangular test samples used are as defined in Figure 2a. The test PSPI samples were fabricated as follows: (a) PECVD (Plasma–enhanced chemical vapor deposition) oxide of 300 nm was deposited on a silicon substrate; (b) PSPI was coated and patterned atop the PECVD oxide layer; (c) the patterned PSPI was fully cured at 300 °C for 1 h; (d) BOE etching was conducted to remove the PECVD oxide; (e) the PSPI strips were completely released from the Si substrate. Figure 2b shows the released PSPI samples.

## 3. Tensile Test of PSPI and FPCB

**Figure 3:**
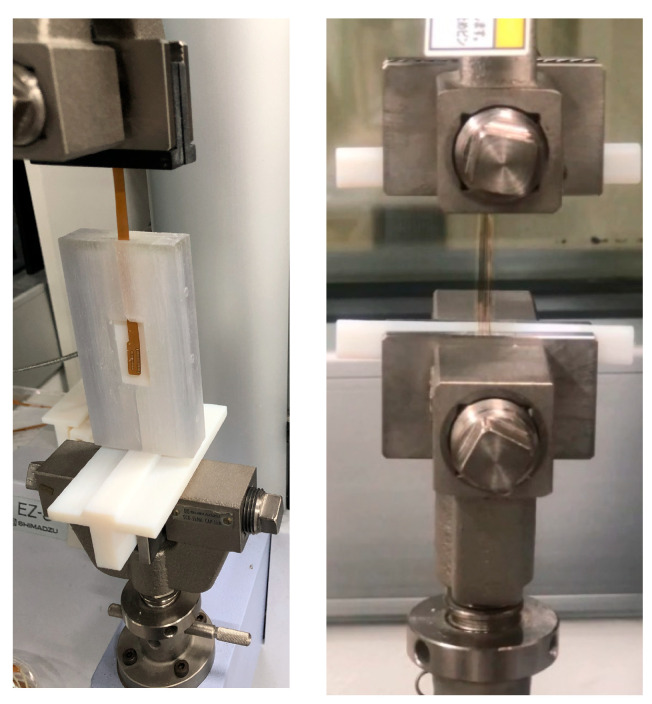
Tensile tests of the FPCB and PSPI test samples.

**Figure 4:**
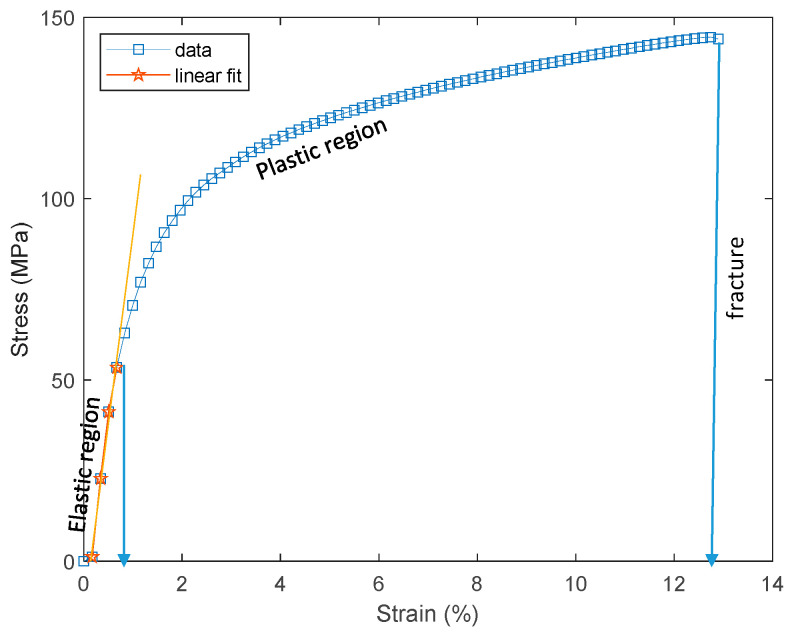
Stress–strain curve of the FPCB test sample.

The PSPI and FPCB test samples were prepared for the tensile tests as follows. The PSPI test samples were 50 mm in length, 5 mm in width, and had two different thicknesses of 5 µm and 25 µm. These dimensions are designed on the basis of the ASTM 882 Standard Test Method for Tensile Properties of Thin Plastic Sheeting. The FPCB test sample was 103.6 mm in length, 4 mm in width, and 200 µm in thickness. The tensile tests were performed with a Shimadzu EZ-S machine (Shimadzu, Kyoto, Japan), as shown in Figure 3. As seen from the figure, the test sample was loaded into the test jig and a displacement was applied to the upper end while fixing the other end. The reaction force was recorded until the test sample was fractured. Figure 4 presents the stress–strain measurements of the FPCB sample. The data presented herein shows the average value of three samples. The stress–strain curve shows that it has two different regions: linear-elastic and plastic. The tensile strength was estimated as 145 MPa when a 13% strain was applied. The slope of the linear-elastic region indicates the elastic modulus of the material, and the yield stress can also be found. The plastic region is used to extract the true stress and true strain values, which are then input to the FEM modeling and simulation. The relationship between the true and measured stresses is shown in Equation (1).

(1) $$\varepsilon_{true}=ln\left(1+\varepsilon_{mea}\right),\cdots \sigma_{true}=\sigma_{mea}\left(1+\varepsilon_{mea}\right)$$

**Figure 5:**
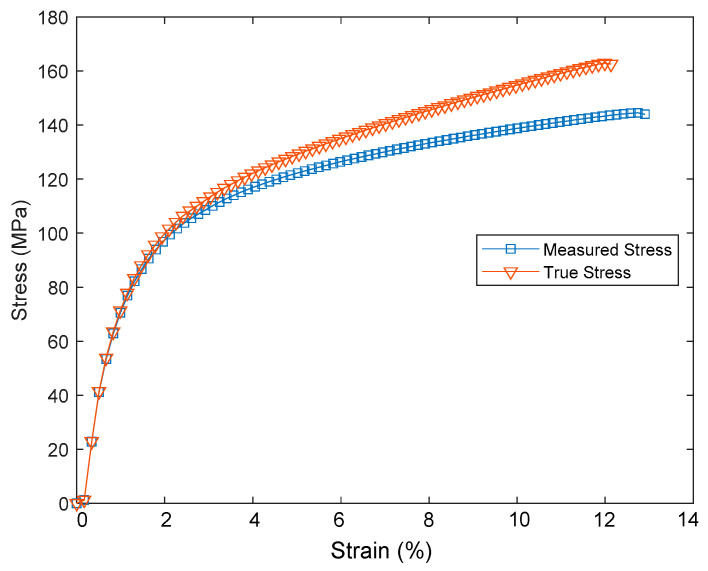
Comparison of measured and true stresses.

Figure 5 shows the comparison of the measured and true stresses as a function of the applied strain. The elastic regions of the two stresses are observed to be matched, while the plastic region of the true stress is greater than that of the measured stress. For the FEM modeling, the plastic strain values can be found using Equation (2).

(2) $$\varepsilon_{plastic}=\varepsilon_{mea}-\sigma_{mea}/E$$

where *E* is the Young’s modulus of the material. It should be noted that all the data are based on the measured stress.

**Figure 6:**
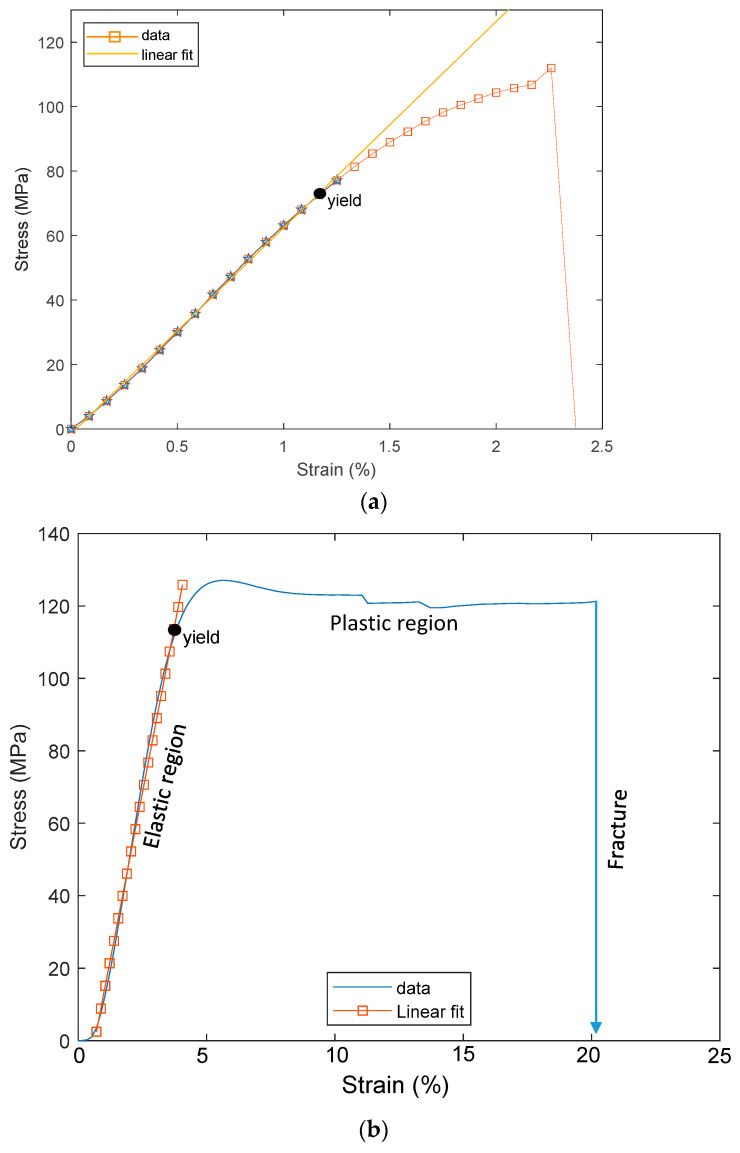
Stress-strain curves of PSPI test samples. (**a**) Thickness = 5 µm. (**b**) Thickness = 25 µm.

Similarly, tensile tests were performed on the PSPI samples. The sample thicknesses of 5 µm and 25 µm were prepared to determine the effects of thickness on the stress–strain curve of the PSPI material. Thin PSPI samples do not show plastic deformations before fracturing at 2.3% applied strain, while thick PSPI samples show relatively wider plastic deformations and fracture when the strain applied reaches 20%, as shown in Figure 6. The PSPI sample of 5 µm thickness is fractured at 110 MPa, while the 25 µm sample is fractured at 120 Mpa. Table 1 summarizes the mechanical properties of the FPCB and PSPI extracted from the stress–strain curves. Note that the plastic region of the PSPI has also been converted using Equations (1) and (2) for FEM modeling in the following section. As shown in Table 2, both the FPCB and PSPI samples show that the fracture locations are located at one end of the sample. In addition, in the fracture region of each sample, irregular fracture shapes were observed.

## 4. FEM Modeling and Simulation

**Figure 7:**
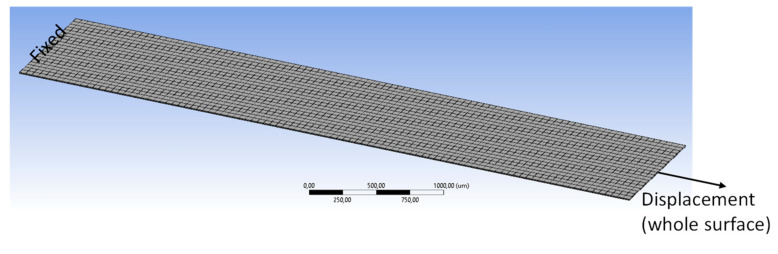
FEM model and its boundary conditions.

**Figure 8:**
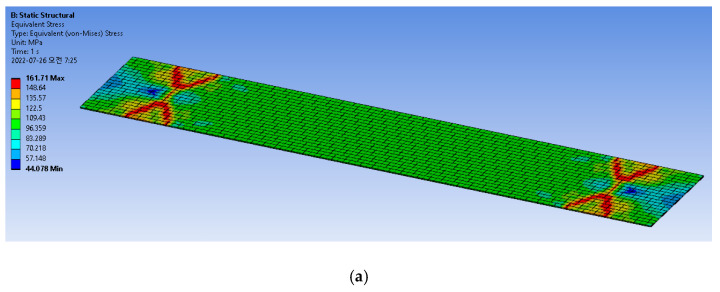

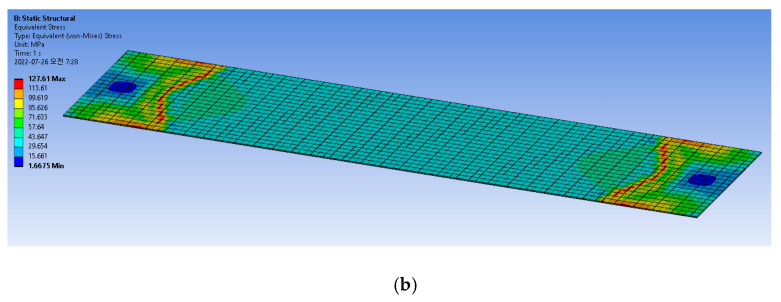
FPCB and PSPI stress distributions during tensile tests. (**a**) FPCB stress distribution when t = 10 µm. (**b**) PSPI stress distribution when t = 10 µm.

The tensile tests were FEM modeled and simulated with the extracted material properties presented in Table 1. Simulations were also performed using the Explicit Dynamics method, which is regarded as more suitable for material failures such as fractures; this method does not need matrix formulation like the conventional simulation, so the simulation time is much shorter. Figure 7 shows the meshed FEM model for the tensile test; the model has dimensions of 5 mm in length and 1 mm in width. A smaller model than that of the experiment was built to reduce simulation time. The number of elements of the FEM model is 120,000. The boundary conditions were as follows: one end of the model was fixed while a displacement load was applied to the other end. The stress distributions of the model during tensile testing for both FPCB and PSPI are presented in Figure 8. The maximum stress is observed near both ends of the test sample or close to the fixed surface and displacement load end, as observed previously in [26]. The maximum stress values are 162 MPa for FPCB and 128 MPa for PSPI, and these correspond to the maximum tensile strength measurements from each material, as shown in the previous section.

**Figure 9:**
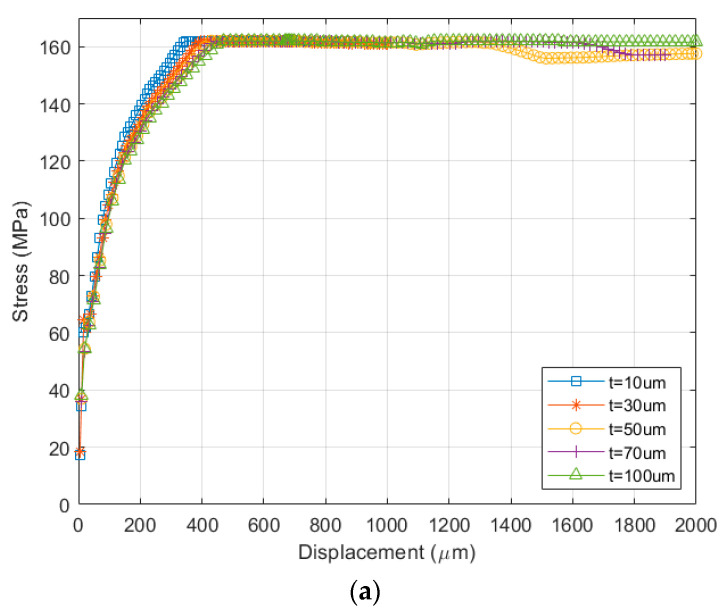

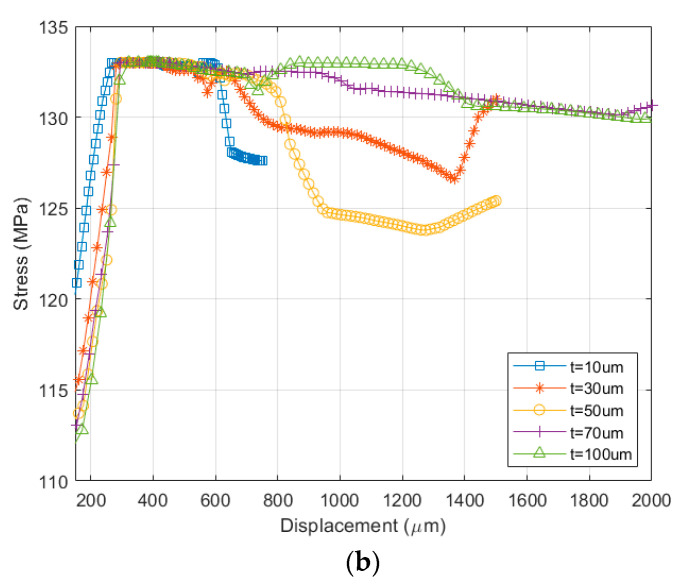
Stress-strain curves depending on the sample thickness. (**a**) FPCB. (**b**) PSPI.

Given these models, parametric simulations were performed to determine the geometric design effects on the stress–strain curve. Figure 9 shows the stress–strain curves for different sample thicknesses of the FPCB and PSPI. The FPCB shows a typical elastic-plastic response, indicating the onset of plastic deformation after the yield point at the end of the linear-elastic region. The PSPI shows necking after the yield point and strain hardening response up to 500 µm. It is known that the neck stabilizes and begins to extend by drawing fresh material from the tapered regions on either side until the entire parallel section of the specimen yields. Then, during alignment and orientation of the polymer chains, which is called strain hardening, the neck continues to taper until it breaks [16]. The PSPI layer thicker than 50 µm has almost the same plastic deformation as that of the FPCB. The maximum stress of the FPCB is estimated as 160 MPa and that of the PSPI is 133 MPa. Therefore, the simulation results are in good agreement with the measurements. It should be noted that the sample of thickness 5 µm is not included in the simulation owing to the convergence problem.

It could be said that a thin PSPI feedthrough may have a larger deformation owing to the strain hardening effect, but there is a thickness limit for this strain hardening effect as the 5-µm-thick PSPI was observed to fracture easily without strain hardening.

**Figure 10:**
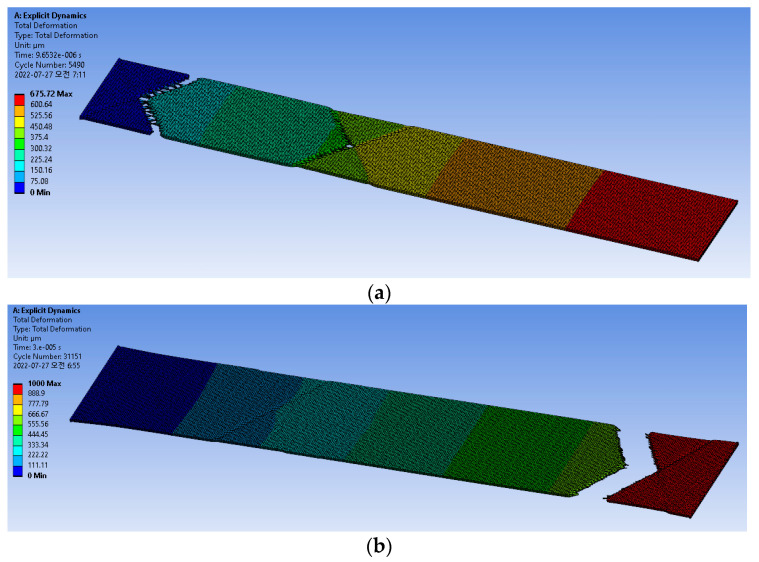
Total deformation by Explicit Dynamics modeling. (**a**) PSPI. (**b**) FPCB.

As fracturing of the tensile test sample is not supported by conventional FEM simulations, the Explicit Dynamics method is used for fracture simulation. The advantages of the Explicit Dynamics method include no matrix formulation and convergence; thus, the simulation time is shorter than that of the conventional approach. This method is generally recommended for problems of extreme nonlinearity and complex material behaviors. For this simulation, the same boundary conditions as shown in Figure 6 were applied. Figure 10 shows the tensile test simulation results with the fracture of the test sample. Note that the fracture occurred near one end of the sample, corresponding with the experimental result. However, the fracture shape in the fracture region may be difficult to determine, similar to that of the experiment result in Table 2; this is believed to have been caused by an error in the axial equilibrium during the loading of the sample into the tensile test machine. In the general setup in the FEM, the axial error (angle or distance) between the axial directions of the tensile force and sample is normally assumed to be 0.

**Figure 11:**
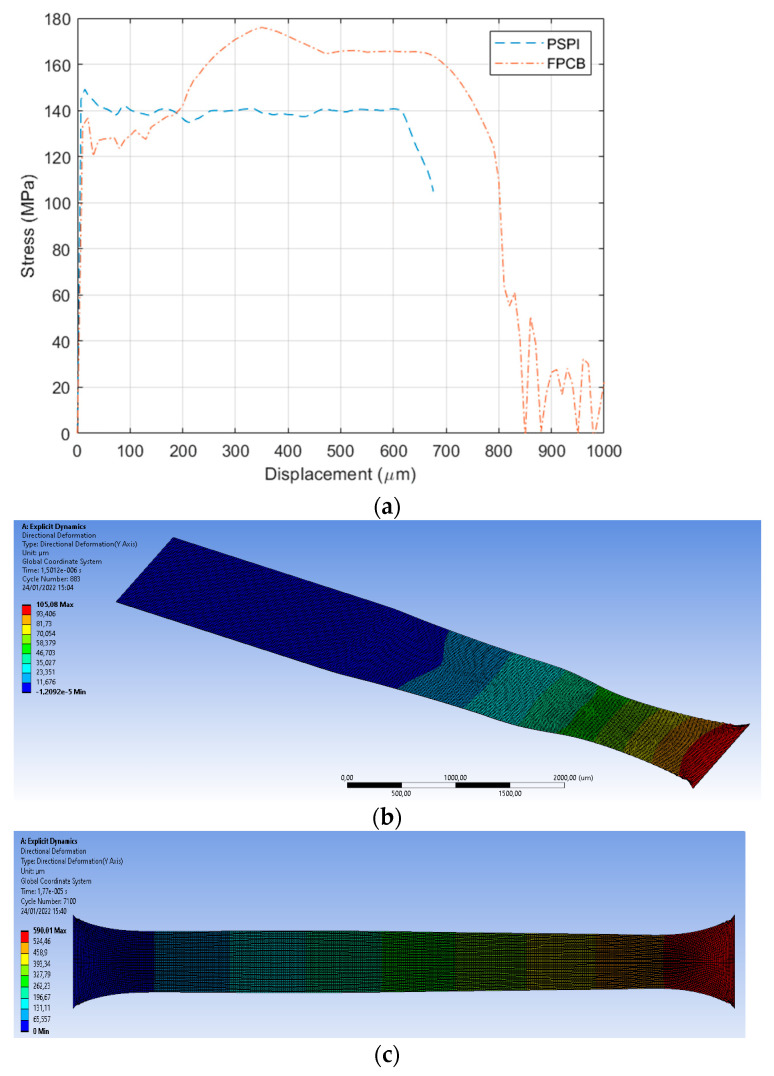
Explicit Dynamics simulation results. (**a**) Stress and displacement curves. (**b**) Necking of PSPI (magnified by 12). (**c**) Necking of FPCB.

The stress–strain curves were extracted at the end of the test sample, as shown in Figure 10a. The PSPI shows strain hardening and necking after the yield point, while the FPCB shows linear-elastic, plastic deformation, and hardening after the plastic region before the fracture. The very difference between the two materials is the portion of the plastic deformation in the stress-displacement curves. Although the PSPI introduces a narrower plastic deformation compared with the FPCB, it has a comparable strain limit before fracture because of the large strain hardening. As shown in Figure 11b,c, the necking effect is more pronounced in PSPI than in FPCB.

**Figure 12:**
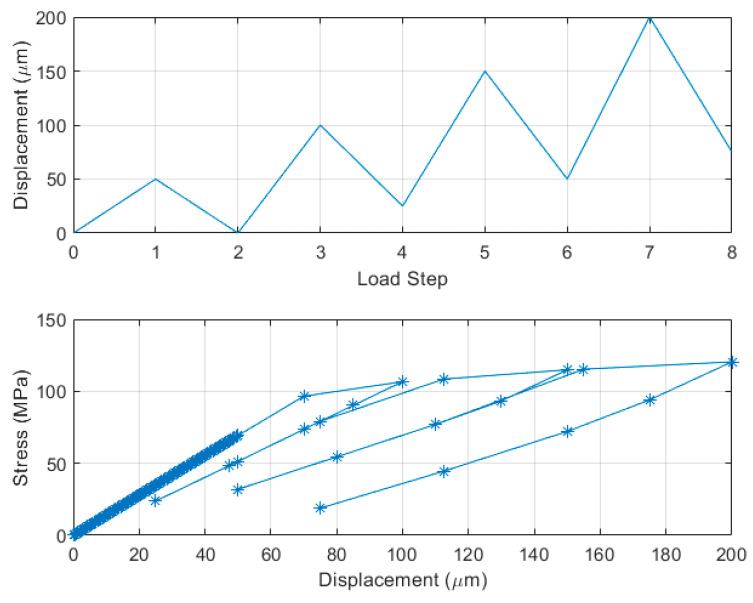
Tensile test of the PSPI model with cyclic loading; loading condition (**top**) stress-displacement curve (**bottom**).

**Figure 13:**
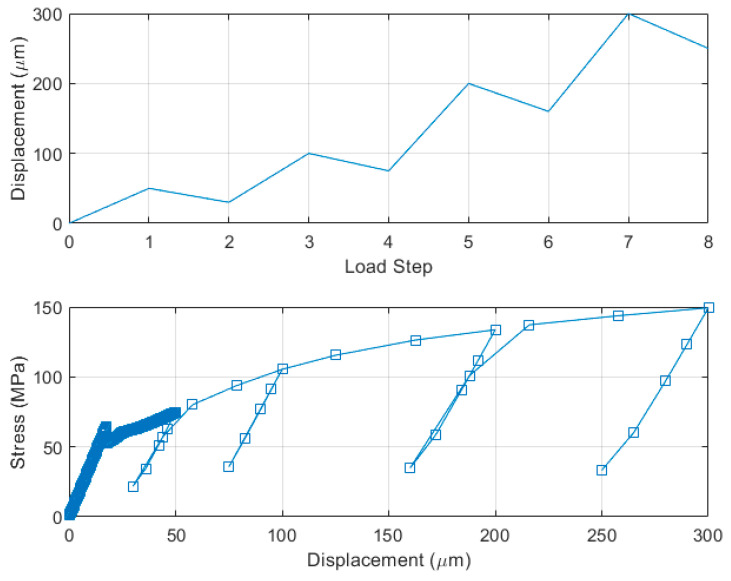
Tensile test of the FPCB model with cyclic loading; loading condition (**top**) stress-displacement curve (**bottom**).

Given the PSPI and FPCB behaviors, a periodic displacement load was applied to the end of the model to study the effects of plasticity on the reliabilities of the materials. Figure 12 shows the PSPI tensile test with a cyclic load; the applied cyclic displacement load is shown in the upper figure. Each cycle is composed of two load steps: the first step is the loading of the displacement, and the second step is unloading. Starting from 50 µm displacement, the applied load is increased in 50 µm steps, up to 200 µm. The new displacement is applied repeatedly after unloading the previously applied displacement. In the first loading composed of steps 1 and 2, linear-elastic behavior was observed as expected. The maximal stress at first loading was 69 MPa, which was far less than the experimental tensile strength of the PSPI. From the second loading, the plastic deformation emerged owing to the larger displacement loading. The maximal stress increased from 69 MPa at first, loading to 120 MPa at the last loading as the peak displacement at each loading increased. From the second loading, the developed stress deviates from the linear-elastic regime owing to the plastic deformation of the material. Unloading of each displacement phase explicitly showed that plastic deformation existed after unloading the displacement load. The plastic deformations were estimated to be 3.6 µm after 100 µm displacement, 12.6 µm after 150 µm displacement, and 55.1 µm after 200 µm displacement. Such plastic deformation would be the principal reason for mechanical failure, frequently mentioned as “fatigue” in the electronic packaging. In the case of cyclic loading of the FPCB, similar displacement loads were applied in the tensile tests, as shown in the upper part of Figure 13. At first, it was observed that the FPCB had a narrower elastic-linear regime than the PSPI. The plastic deformation of the FPCB started from 25 µm axial displacement, while that of the PSPI was estimated to start from 75 µm. The maximal stress at first loading was approximately 75 MPa, which was larger than that of the PSPI. It should be noted that the maximal stress of the FPCB includes two components, namely elastic stress, and plastic stress. The maximal stress of the FPCB was estimated as 150 MPa at 300 µm displacement loading, which was close to the experimental tensile strength of 140 MPa. The plastic deformations were estimated as 22.4 µm after 50 µm displacement, 62.4 µm after 100 µm displacement, 147.3 µm after 200 µm displacement, and 237.5 µm after 300 µm displacement. The plastic deformation of the FPCB was substantially larger than that of the PSPI as it produced 62.4 µm (3.6 µm for PSPI) at 100 µm of applied displacement. This larger plastic deformation was attributed to the larger elastic modulus of the FPCB than that of the PSPI as the unloading curve had the same slope as that of the elastic regime after plastic deformation.

## 5. Conclusions

The mechanical behaviors of frequently used polyimide feedthroughs were studied by tensile tests and FEM simulations for implantable biocompatible packages. The tensile test measurements reveal that the PSPI feedthroughs have larger strain ranges compared with those of the FPCB owing to the strain hardening effect, and the mechanical properties of the PSPI feedthroughs depend on the thickness. Interestingly, the yield strengths of the PSPI feedthroughs and FPCB are quite similar although the thickness differences are substantial. FEM modeling and simulation with the experimentally extracted material properties confirm that the strain hardening effect of the PSPI is the principal cause of the large strain operation of the material. In addition, cyclic loading of the displacement shows that the plastic deformation is dominant when the input displacement is beyond the linear-elastic regime, which can create material failure due to fatigue of the material.

## Figures and Tables

**Table 1:** Summary of FPCB and PSPI mechanical properties.

Samples	Sample Dimension	Elastic Modulus (GPa)	Yield Stress (MPa)	Tensile Strength (MPa)	Tensile Strain (%)
PSPI I	L = 50 mm W = 5 mm T = 5 µm	6.3	110	110	2.39
PSPI II	L = 50 mm W = 5 mm T = 25 µm	3.68	125	120	20
FPCB	L = 103.6 mm W = 4 mm T = 200 µm	10.5	70	140	12.9

**Table 2:** Result images of tensile tests with FPCB and PSPI samples.

	Sample Types	FPCB	PSPI
Images			
After Tensile Test		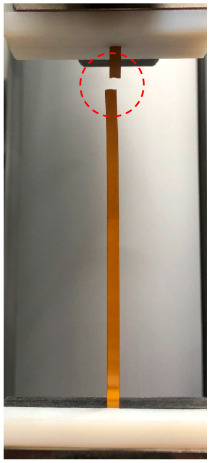	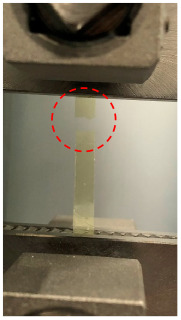
Microscopic Image of Fractured Region